# Hsp31, a member of the DJ-1 superfamily, is a multitasking stress responder with chaperone activity

**DOI:** 10.1080/19336896.2016.1141858

**Published:** 2016-04-20

**Authors:** Kiran Aslam, Tony R. Hazbun

**Affiliations:** Department of Medicinal Chemistry and Molecular Pharmacology and the Purdue University Center for Cancer Research, Purdue University, West Lafayette, IN, USA

**Keywords:** Hsp31, DJ-1 superfamily, stress response, chaperone, methylglyoxalase, degylcase, prion, α-synuclein

## Abstract

Among different types of protein aggregation, amyloids are a biochemically well characterized state of protein aggregation that are associated with a large number of neurodegenerative diseases including Parkinson's disease, Alzheimer and Creutzfeldt-Jakob disease. Yeast, *Saccharomyces cerevisiae* is an insightful model to understand the underlying mechanism of protein aggregation. Many yeast molecular chaperones can modulate aggregation and misfolding of proteins including α-Syn and the Sup35 prion. Hsp31 is a homodimeric protein structurally similar to human DJ-1, a Parkinson's disease-linked protein, and both are members of the DJ-1/ThiJ/PfpI superfamily. An emerging view is that Hsp31 and its associated superfamily members each have divergent multitasking functions that have the common theme of responding and managing various types of cellular stress. Hsp31 has several biochemical activities including chaperone and detoxifying enzyme activities that modulate at various points of a stress pathway such as toxicity associated with protein misfolding. However, we have shown the protective role of Hsp31's chaperone activity can operate independent of detoxifying enzyme activities in preventing the early stages of protein aggregate formation and associated cellular toxicities. We provide additional data that collectively supports the multiple functional roles that can be accomplished independent of each other. We present data indicating Hsp31 purified from yeast is more active compared to expression and purification from *E. coli* suggesting that posttranslational modifications could be important for Hsp31 to be fully active. We also compare the similarities and differences in activities among paralogs of Hsp31 supporting a model in which this protein family has overlapping but diverging roles in responding to various sources of cellular stresses.

## INTRODUCTION

Parkinson's disease is associated with progressive deterioration of dopaminergic neurons in the *substantia nigra* and the second most common age-related neurodegenerative disease.^1^ Accumulation of high level of reactive oxygen species (ROS), mitochondrial dysfunction and α-Syn aggregation are forms of cellular stress that lead to toxicity and neuronal cell death.^2^ Mutations in certain genes are involved in the development of familial form of PD including the PARK7 gene encoding DJ-1. DJ-1 is a member of ThiJ/DJ-1/PfpI protein superfamily that are a quintessential multitasking or moonlighting protein family as evidenced by their involvement in multiple cellular functions including oxidative stress sensing, protein folding, proteasome degradation, mitochondrial complex stabilization, methylglyoxalase and deglycation enzyme activities.^3-7^ The *E. coli* protein, hchA, has been bioinformatically identified as a moonlighting protein.^8^ The members of the ThiJ/DJ-1/Pfp1 superfamily appear to have evolved to numerous mechanisms to manage cellular stress. The protein superfamily members are present across the evolutionary spectrum including prokaryotes and the budding yeast, *Saccharomyces cerevisiae*, that has four paralogs named Hsp31, Hsp32, Hsp33 and Hsp34.^9,10^ Hsp31 consists of 237 amino acids with a molecular weight of 25.5 kDa and forms a homodimer in solution.^9^ It possesses the Cys-His-Glu catalytic triad common to ThiJ/DJ-1/PfpI superfamily proteins.^9,10^ This short review will discuss recent studies demonstrating Hsp31 exhibits diverse cellular functions to assist cellular survival under stress conditions.

## ROLE OF HSP31 IN CELLULAR STRESS RESPONSE AND PROTEOTOXIC STRESS

Consistent with the main role of cellular stress response, Hsp31 expression is strongly induced during late phases of growth and required for survival under conditions of nutrient limitation.^11^ Hsp31 expression is also induced when yeast cells are treated with H_2_O_2_ to produce oxidative stress and this up-regulation of Hsp31 is under the control of stress responsive transcription factor, Yap1.^12,13^ In addition, Hsp31 possesses robust glutathione independent glyoxalase activity that converts the toxic metabolite, glyoxal (MG), into D-lactate.^12,14,15^ The catalytic triad Cys-His-Glu of Hsp31 is vital for this enzymatic activity and critical for suppressing the elevated level of ROS by MG.^12,15^ An overall view has emerged that indicates Hsp31 has important metabolic and regulatory roles in cytoprotective pathways.^16^

**FIGURE 1.**
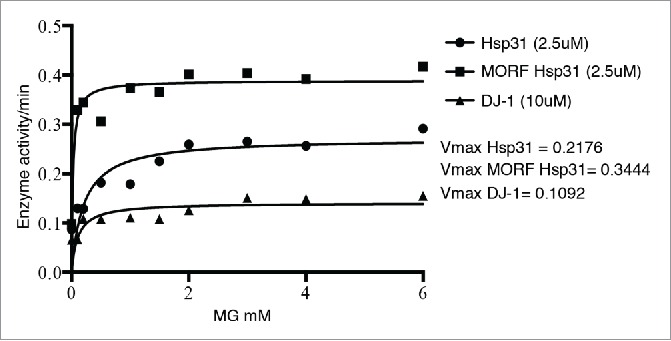
The hyperbolic plot of substrate concentration versus rate of D-lactate production by Hsp31 (circles), MORF Hsp31 (squares) and DJ-1 (triangles) are shown and the solid line represents the Michaelis-Menten best-fit model. The Vmax parameters were determined based on this model.

In addition to the enzyme activity, we also demonstrated that Hsp31 has broad chaperone activity in several classic protein aggregation assays indicating its ability to manage misfolded proteins that initiate proteotoxic stress in the cell.^12^ Intriguingly, Hsp31 purified directly from a yeast expression system, the GAL promoter induced movable ORF tag system (MORF),^17^ had increased enzymatic activity compared to Hsp31 purified from recombinant *E. coli* (Fig. 1). We also demonstrated that the yeast-purified protein was more active in preventing aggregation of several substrate proteins including α-Syn. The increased activity may be due to the difference in the affinity tags used but may be a result of posttranslational modification(s) that occur in the cell. Several reports have indicated that post-translational modifications or differing levels of oxidation of the cysteine residue can alter activity of DJ-1.^18,19^ We also show that Hsp31 is a more potent methylglyoxalase compared to DJ-1 consistent with several other studies (Fig. 1).^14,15^ In addition, we showed that yeast Hsp31 is more active in preventing protein aggregation compared to DJ-1. Interestingly, it has also been observed that Hsp31 rescues α-Syn toxicity to a greater extent than DJ-1 *in vivo*.^5^ These results raise the intriguing possibility that Hsp31 is constitutively active whereas DJ-1 must undergo an activation event to increase its activity.

Hsp31 inhibits the formation of aggregates of a wide range of proteins including insulin, citrate synthase, α-Syn and the Sup35 prion.^12^ The recombinant soluble α-Syn and Sup35 proteins can readily polymerize into amyloidogenic fibrils *in vitro*.^20^ Hsp31 inhibits protein aggregation formation *in vitro* and foci formation of GFP-tagged proteins *in vivo*. In addition, over-expression of Hsp31 can rescue cells from toxicity associated with overexpression of α-Syn.^12^ The rescue phenotype could be mediated by several different mechanisms including Hsp31's methylglyoxalase or deglycation activities. However, a Hsp31 mutant deficient in methylglyoxalase activity is still active in preventing α-Syn *in vitro* aggregation and prevention of toxicity from overexpressed α-Syn. Another possible rescue mechanism could be the autophagy pathway, particularly because deletion of *HSP31* impairs autophagy under carbon starvation conditions.^11^ Autophagy does alleviate toxicity from α-Syn overexpression because we show a synthetic lethal relationship between α-Syn overexpression and deletion of *ATG8*. However, we demonstrated that overexpression of Hsp31 in the *atg8*Δ strain can rescue α-Syn-mediated toxicity.^12^ These data show that despite the multitasking abilities and roles of Hsp31, the chaperone activity appears to have the ability to prevent α-Syn independent of other activities. In support of our finding, another study found the autophagy pathway was not crucial in preventing the Hsp31 chaperone activity against a cytoplasmic aggregation prone protein.^21^ The same study also demonstrated that Hsp31 chaperone activity overlaps with the Ubr-dependent degradation pathway but is independent of its function oxidative stress response.^21^ Further exploration of this model would need to utilize mutants that abrogate chaperone function without affecting enzyme activity and other biological functions.

**FIGURE 2.**
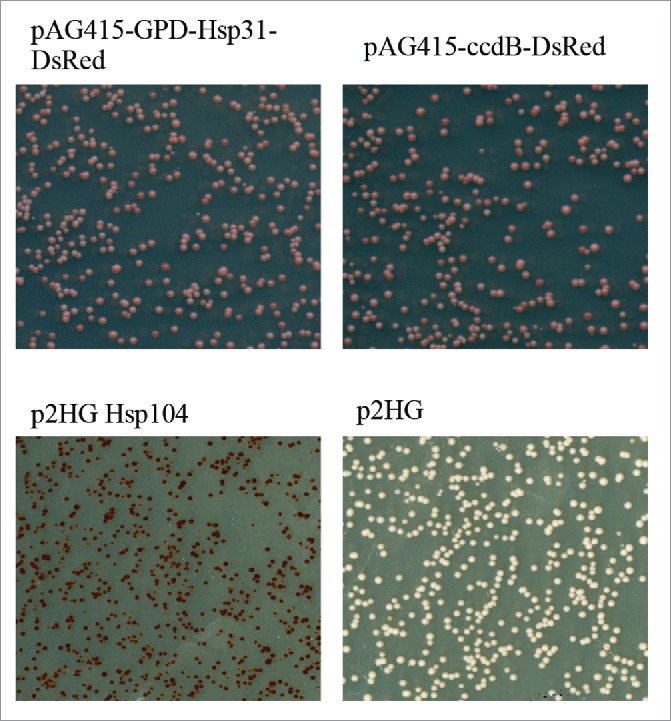
Hsp31 cannot resolve the Sup35 amyloids. [*PSI*+] cells were transferred with pAG415-GPD-Hsp31-DsRed, p2HG Hsp104 and their corresponding empty vectors. Cultures were incubated at 30°C for 16 hrs before plating on ¼ YPD medium to develop colony color. Change from white to red color phenotype demonstrated curing. Hsp104 is able to cure the [*PSI*+] phenotype as seen by the red colonies. Hsp31 expression under these conditions can transiently reduce prion aggregation as shown by semi-denaturing gels and microscopy but is not able to cure prions.

The typical models of protein aggregation propose unfolded monomers as an initiating event that progresses to unstable oligomeric intermediates, and finally elongates to larger oligomers. The observed anti-aggregation activity of Hsp31 raises the question of what stage Hsp31 intervenes in the protein aggregation process. On the bases of our *in vitro* studies, Hsp31 likely interacts with early oligomeric intermediates of α-Syn and therefore prevents higher oligomer formation. For example, when soluble α-Syn was mixed with Hsp31, formation of precipitated SDS-resistant oligomers was markedly reduced. Likewise, the soluble fraction of α-Syn, which included SDS-resistant oligomers in the size range of 25-50 KDa, was almost completely abolished in the presence of Hsp31 indicating that Hsp31 likely interacts with the monomeric or early oligomers in preventing the formation of higher order oligomers. We observed similar results with the *in vitro* ThioT assay, in which incubating the Hsp31 with α-Syn completely prevented the increase in fluorescence intensity associated with increasing fibril formation again supporting our model that Hsp31 acts at early stages of protein aggregation.^12^ Of particular interest is that DJ-1 has been shown to interact with monomeric and oligomeric forms of α-Syn as determined by co-IP.^5^ This same study also showed that DJ-1 interacts with α-Syn *in vivo* as well. Along with α-Syn aggregation formation, we also demonstrated the inhibitory effect of Hsp31 on the Sup35 prion based on reduction of aggregates observed in cell lysates. These results support the notion that Hsp31 acts at the early stages of oligomerization to prevent further protein oligomerization.^12^ Interestingly, we previously showed that the overexpression of Hsp31 reduces the level of Sup35 aggregation but we also show that it is unable to cure prions from a [*PSI*^+^] strain (Fig. 2). [*PSI*^+^] strains contain Sup35 prion aggregates that can be cured by chaperones such as Hsp104, but the lack of curing by Hsp31 suggests that it lacks disaggregase activity and cannot intervene in an established prion cycle. Moreover, our study showed a lack of co-localization between Hsp31 and Sup35 aggregates with fluorescence microscopy, rather Hsp31 is occluded from Sup35 prion aggregates, indicating that Hsp31 acts on its substrates prior to the formation of large aggregates.^12^ The strong chaperone activity of Hsp31 suggests that it may modulate prion aggregates but might need to cooperate with other chaperones similar to what has been demonstrated with other small HSPs such as Hsp26 and Hsp42.^22^ Further studies investigating the role of Hsp31 in modulating prionogenesis are ongoing in our lab.

## COMPARISON OF HSP31 PARALOGS

The Hsp31 mini-family is composed of four paralogs; Hsp31 (*YDR533C*), Hsp32 (*YMR322C*), Hsp33 (*YOR391C*), and Hsp34 (*YPL280W*).^9,10^ Genes of the Hsp31 mini family are located at the subtelomeric region of the genome in *Saccharomyces cerevisiae*. HSP31 is considered the parental gene with HSP32, HSP33 and HSP34 originating from it during gene duplication events. Among all the members of this mini-family, Hsp31 is most divergent and it shares approximately 70% homology with the other members of the family those possess more than 90% homology between them.^9^ All the members of Hsp31 family contain the same Cys-His-Glu catalytic triad as present in the *E. coli* ortholog but interestingly no protease activity has been detected so far for Hsp31 or other paralogs. Previously, it was shown that mutation in the catalytic triad largely abolishes glyoxalase activity^12,15^ but this catalytic triad is not required for chaperone activity of Hsp31.^12^ These results indicate that the anti-aggregation activity of Hsp31 is not under the influence of its enzymatic activity rather, it has a direct chaperone activity against misfolded proteins. Intriguingly, all the paralogs of the Hsp31 minifamily possess comparable activity against α-Syn aggregation and toxicity when they are overexpressed from the *GAL* promoter.^5^ Furthermore, the chaperone activity of Hsp31, Hsp32 and Hsp33 against a cytoplasmic aggregation-prone protein is independent of their role in oxidative stress response and the vacuolar degradation pathway.^21^ However, unlike Hsp31, the other paralogs possess very little methylglyoxalase activity and are unable to protect the cells from glyoxal toxicity.^15^ These results again support the notion that anti-aggregation activity of Hsp31 mini-family is independent of its enzymatic activity. In addition, the lack of methylglyoxalase activity in the paralogs is evidence that the paralogs are diverging but additional studies dissecting the roles within this paralog group are needed to further uncover these diverging functions. The Hsp31 protein family are broadly spread across fungal species with varying levels of paralog duplications and additional evidence of divergence including differences in localization in the *Schizosaccharomyces pombe* Hsp31 family members.^23^ A functional comparison of Hsp31 and its paralogs are summarized in Table 1 highlighting the similarities and differences among these proteins.

**TABLE 1.** Functional summary of Hsp31 and paralogs.

Function/Attribute	Hsp31	Hsp32	Hsp33	Hsp34
Catalytic triad	Yes	Yes	Yes	Yes
Chromosome Position	Interstitial	Telomeric	Telomeric	Telomeric
Sequence homology	˜70%	>90%	>90%	>90%
Chaperone activity	+++	+++	+++	+++
Methylglyoxalase	+++	+/−	+/−	+/−
Deglycase	+++	ND	ND	ND
Role in Autophagy	+	+	+	+
Peak mRNA level	Early SP	DS	Early SP	ND
Peak steady state protein level	SP	ND	ND	ND
Stress granule and P body localization	Yes	Yes	ND	ND
Mitochondrial localization	Yes	ND	ND	ND

ND = Not determined

SP = Stationary Phase

DS = Diauxic shift

## HSP31 PLAYS AN IMPORTANT ROLE IN MAINTAINING REDOX HOMEOSTASIS

Oxidative stress occurs when intra-cellular reactive oxygen species (ROS) overwhelms the anti-oxidative defense system in the cell present during normal aerobic metabolism or by exposure to external radical generating agents. ROS triggers damage to macromolecules in the form of oxidative modifications and misfolding of proteins, associated with the development of diseases and pathological conditions such as PD and prions.^2,24,25^ Hsp31 has an important role in maintenance of redox homeostasis in yeast under oxidative stress generated by methylglyoxal or H_2_O_2_.^13,15^ Like many other heat-shock genes *HSP26, HSP12, HSP82*, and *SSA3*, expression of *HSP31* is strongly induced at diauxic shift when the cells are stressed by nutrient limitation and by accumulation of oxidative metabolites.^11-13^ Others and we also reported an elevated level of Hsp31 under oxidative stress when cells were treated with H_2_O_2_.^12,13^ Similarly, Hsp31 also plays a role in the survival of cells during stationary phase and protects cells from oxidative stress caused by methylglyoxal and H_2_O_2_ accumulation.^13,15^ In addition, we demonstrated that Hsp31 expression was induced under proteotoxic stress such as overexpression of α-Syn. In support of the role of Hsp31 in managing this proteotoxic stress, we found that deletion of *HSP31* synergizes with α-Syn expression to increasing toxicity. We reported an increase in ROS level in the *hsp31*Δ strain that correlates with increased toxicity by α-Syn expression compared to wild type strains, indicating that the presence of Hsp31 is important in reducing ROS to basal levels.^12^ In agreement with our study, overexpression of Hsp31 robustly suppresses both cytosolic and mitochondrial ROS levels instigated by MG and H_2_O_2_ and therefore provides cytoprotection.^15^ In addition, Hsp31 localizes to mitochondria and preserves mitochondrial integrity by redistributing glutathione to the cytoplasm under oxidative stress.^15^ Another study examined the deglycase activity of Hsp31 and showed that it efficiently deglycates proteins with glycated Cys, Arg and Lys amino acid residues.^26^ Taken together, these results suggest that Hsp31 is an integral part of the heat shock protein system and plays a vital role in maintaining cellular homeostasis.

## CONCLUSION

**FIGURE 3.**
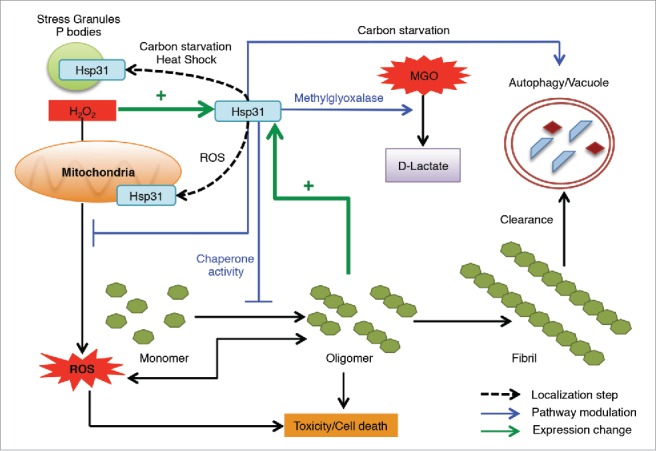
The homeostatic functions of Hsp31 associated with protecting cells from stress. Hsp31 is a methylglyoxalase that converts MGO into D-lactate independent of glutathione. Proteotoxic stress induced the expression of Hsp31, which exerts a protective function against toxic effect of oligomers in yeast cells. Oxidative stress induces the expression of Hsp31, re-localizes it to mitochondria resulting in reduced levels of ROS. Response to other stresses leads to Hsp31 localization to P bodies and stress granules. *HSP31* deletion under carbon starvation compromises the autophagy pathway, which is a pathway used to clear oligomerized or aggregated proteins. Despite the role of Hsp31 in autophagy, it has a protective effect against α-Syn oligomerization independent of its role in autophagy because of its inhibitory effect early in the oligomerization process.

Once again, our results together with other recent findings demonstrate the multitasking ability of Hsp31, which is particularly important during stressful situations. It functions as a stress response chaperone, glutathione independent methylglyoxalase, has a role in the autophagy pathway and acts as a deglycase. Other possible functions have been observed for this superfamily including a report of RNA binding for DJ-1 and protease activity for other family members.^27-30^ These multiple functions can modulate the protein misfolding and stress pathways at various points in the cellular network but our results also highlight that Hsp31 has the ability to inhibit protein aggregation distinct of its enzymatic activity (Fig. 3). We believe that Hsp31 acts at the initial phases of protein misfolding process and prevents the formation of larger aggregates but does not possess disaggregase activity.

## FUTURE DIRECTIONS

Given the apparent functional diversity of Hsp31 revealed so far, it is likely that there might be other chaperone dependent and independent functions of this protein that may exist. Many heat shock proteins (HSP) work in collaboration with other chaperone in order to be fully active. The exploration and identification of protein-protein interaction partners of Hsp31 would provide insight on mechanism and roles of Hsp31. There is dearth of protein-protein interaction information for Hsp31 although it was reported to interact with other chaperones according to a large-scale proteomic study.^31^ Deletion of *HSP31* down-regulates the Ssa3, a Hsp70 paralogn, mRNA level at stationary phase suggesting a correlation between Hsp31 and Hsp70 activity.^11^ Human homolog DJ-1 is known to interact with many chaperones including Hsp70 and mitochondrial Hsp70 indicating that translocation of DJ-1 to mitochondria depends on these chaperones.^4^ A distinct possibility is that relocation of Hsp31 to mitochondria, P bodies or stress granules under oxidative stress is dependent on interactions with other chaperones. Our data support that Hsp31 acts at early stages of protein aggregation but the precise mechanism is not clear. Hsp31 may interact with unfolded monomers to sequester them from progressing to oligomers or alternatively, it might become active only after smaller oligomers are formed. The experiments we performed do not differentiate between these two scenarios and therefore further investigation is needed. Although we have shown that Hsp31 cannot cure [*PSI*^+^] prions, its ability to reduce the Sup35 prion aggregation raises the possibility that Hsp31 has a potential role in modulating prion induction.

## Abbreviations

α-Syn: α-Synuclein
PD: Parkinson's disease
HSP: Heat shock protein
ROS: Reactive oxygen species
MGO: Methylglyoxal
MORF: Yeast movable ORF
DsRed: Red fluorescence protein from Discosoma sp
